# Lightweight Stereo Vision for Obstacle Detection and Range Estimation in Micro-Mobility Vehicles

**DOI:** 10.3390/s26061988

**Published:** 2026-03-23

**Authors:** Jiansheng Ruan, Hui Weng, Zhaojun Yuan, Guangyuan Jin, Liang Zhou

**Affiliations:** School of Mechatronics Engineering, Harbin Institute of Technology, Harbin 150001, China

**Keywords:** micro-mobility vehicles, stereo vision, stereo matching, lightweight networks, range estimation, embedded deployment

## Abstract

Micro-mobility vehicles operating in closed, low-speed environments (e.g., parks) require reliable obstacle detection and accurate range estimation under strict constraints on cost, power, and onboard computation. This paper proposes HAGVNet, a lightweight stereo matching network for embedded ranging and validates its practical deployability in a target-level ranging pipeline with YOLO11n as the front-end detector. HAGVNet builds a hierarchical attention-guided cost volume (HAGV) that uses coarse-scale geometric priors to modulate fine-scale cost modeling and adopts ConvNeXtV2-style 2D cost aggregation blocks to improve stability and boundary consistency with controlled complexity. For ranging, depth statistics within detected regions are used to estimate target distance and 3D position. The model is pre-trained on SceneFlow and evaluated on KITTI. On SceneFlow, HAGVNet reaches 0.73 px EPE with 20.08 G FLOPs, indicating a favorable accuracy–complexity trade-off under low computation budgets. On an embedded Jetson Orin Nano Super platform, HAGVNet achieves 46.3 FPS under TensorRT FP16, and field tests indicate relative ranging errors of 0.5–8.6% within 2–10 m, demonstrating its practical feasibility for low-speed target-level ranging.

## 1. Introduction

With the increasing adoption of micro-mobility vehicles in closed, low-speed environments (e.g., parks), introducing a basic level of autonomous capability can improve on-demand mobility services. A key prerequisite is safe and reliable environmental perception, where the most fundamental capability is stable obstacle detection with trustworthy range estimation [1]. Unlike the high-cost sensing stacks commonly used in automotive systems, micro-mobility platforms are typically constrained by cost, power, and computation, motivating lightweight and deployable ranging solutions on embedded hardware. In this work, micro-mobility serves as a practical testbed for studying embedded stereo-based obstacle ranging in low-speed environments, where the trade-off between accuracy, efficiency, and deployability is particularly important.

Common ranging sensors include LiDAR, ultrasonic sensors, and cameras. LiDAR offers high accuracy but is often expensive and power-hungry, while ultrasonic sensors are inexpensive but provide limited information. Cameras are low-cost and information-rich, making them well suited to lightweight platforms [2]. In vision-based ranging, monocular depth typically relies on motion cues or learned priors and may not provide an absolute metric scale, whereas stereo vision can recover metric depth from disparity under calibration and rectification, with strong interpretability and engineering practicality [3].

Stereo ranging critically depends on stereo matching. Traditional methods (e.g., block matching and SGM) are explainable and training-free but can be fragile under weak texture, occlusion, and challenging illumination [4,5]. Deep stereo matching improves performance in difficult regions via feature learning, cost modeling, and disparity regression [6], yet high-accuracy models are often computationally heavy and unsuitable for embedded deployment. Recent real-time and lightweight models have emerged [7] (e.g., StereoNet [8], AANet [9], MobileStereoNet-2D [10], HITNet [11], Fast-ACVNet [12], and IINet [13]); however, under tight efficiency constraints, multi-scale geometric information is still not fully exploited in cost volume construction, and the guidance of coarse-scale geometric priors for fine-scale matching remains underexplored, particularly in weak-texture and occluded regions.

**Figure 1:**
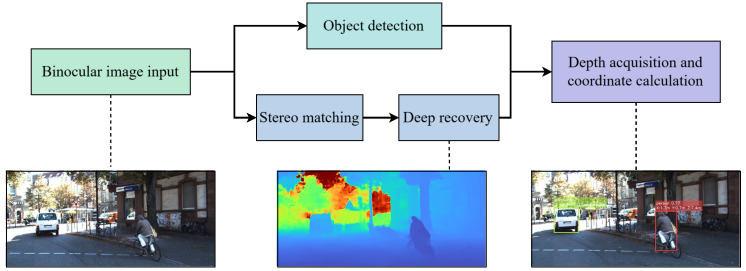
The pipeline of object detection and range estimation based on lightweight stereo vision. The input is a rectified binocular image pair; object detection provides ROIs for target-level depth fusion, while stereo matching estimates dense disparity from the binocular images.

To address these challenges in closed, low-speed micro-mobility scenarios, this paper proposes a lightweight stereo matching network, HAGVNet, and validates its deployability in a target-level ranging pipeline of object detection–stereo matching–depth fusion, as illustrated in Figure 1. The pipeline takes a rectified binocular image pair as input. The methodological contributions focus on stereo matching and depth fusion ranging, while object detection is implemented with YOLO11n as the front-end on the left image. HAGVNet introduces a hierarchical attention-guided cost volume (HAGV) that uses coarse-scale disparity to form geometric priors and modulate fine-scale cost distributions, improving geometric consistency and matching stability. In addition, ConvNeXtV2-style 2D cost aggregation blocks are adopted to strengthen aggregation capacity with controlled complexity. For obstacle perception, YOLO11n outputs bounding boxes [14], which are fused with stereo depth; depth statistics within each detected region are then used to estimate target distance and 3D position, enabling real-time ranging on embedded platforms. The detection branch does not directly supervise stereo matching; instead, it constrains the downstream depth fusion stage by providing target-level ROIs for ranging.

## 2. Lightweight Stereo Matching Model

This section presents the overall framework and key modules of the proposed lightweight stereo matching network, HAGVNet. The design targets real-time depth perception on embedded platforms, aiming to achieve efficient and robust disparity inference under limited computation and memory.

**Figure 2:**
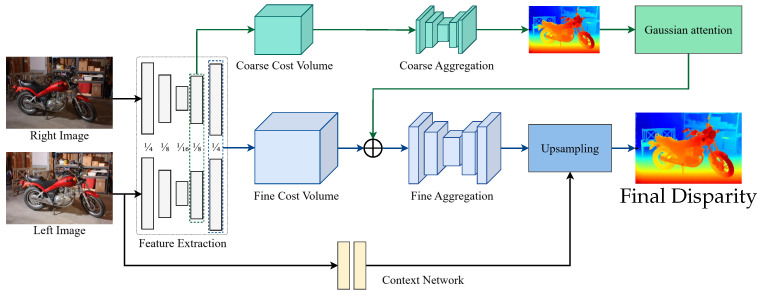
Overview of proposed HAGVNet architecture.

Existing lightweight stereo matching studies mainly reduce the computational burden of cost volume construction and aggregation: StereoNet estimates coarse-to-fine disparity using low-resolution matching and progressive refinement [8]; AANet dynamically modulates multi-scale cost fusion via adaptive aggregation [9]; MobileNetStereo-2D compresses computation using a lightweight backbone and 2D aggregation [10]; HITNet reduces reliance on heavy explicit regularization by hypothesis-driven iterative refinement [11]; Fast-ACVNet and IINet improve information efficiency via efficient cost volume modeling/aggregation and enhanced left–right feature interaction, respectively [12,13]. Despite these advances, under lightweight constraints, the use of multi-scale geometric cues during cost volume construction remains limited. Motivated by this, HAGVNet builds a hierarchical attention-guided cost volume (HAGV) that uses coarse-scale geometric priors to modulate fine-scale cost modeling and combines it with lightweight ConvNeXtV2-style 2D cost aggregation for end-to-end stereo matching. The overall architecture is illustrated in Figure 2.

### 2.1. Hierarchical Attention-Guided Cost Volume (HAGV)

Instead of constructing a cost volume at a single scale, HAGV explicitly introduces a coarse-scale geometric prior to modulate the fine-scale cost volume. Many lightweight stereo models build correlation-based cost volumes only from single-scale features, without constraints from coarse-scale geometry; this can lead to unstable cost distributions in weak-texture, repetitive-texture, and occluded regions. To improve the stability and geometric consistency of fine-scale matching, we propose a hierarchical attention-guided cost volume (HAGV) construction strategy. The key idea is to first obtain a stable geometric prior at a coarse scale and use it as a weak geometric constraint during fine-scale cost modeling so that the fine-scale cost volume becomes geometry-aware at generation time rather than relying only on later aggregation to compensate errors.

Concretely, multi-scale features are extracted from a backbone network. Features at 1/8 and 1/4 of the input resolution are used to build coarse- and fine-scale cost volumes, respectively. The coarse cost volume is aggregated and regressed to obtain a coarse disparity map, which is then upsampled to the fine scale and used to generate a smooth Gaussian attention prior. Here, μ denotes the local mean centered at ${\hat{d}}_{\mathrm{coarse}}$, and σ controls the spread of the Gaussian prior over disparity hypotheses *d*:

(1) $$A\left(d\right)=\exp\left(-\frac{{\left(d-\mu \right)}^{2}}{2\sigma^{2}}\right)$$

The attention prior emphasizes geometrically reliable regions and suppresses uncertain ones. Let $C_{f}$ denote the fine-scale cost volume, and let *A* denote the attention prior. We modulate the fine-scale cost via an additive residual fusion:

(2) $$C_{f}^{\prime }=C_{f}+A⊙\Delta C$$

Here, ⊙ denotes element-wise multiplication, and ΔC is predicted by a learnable 3×3 convolutional residual branch on fine-scale cost features. No hand-crafted scalar is used to mix coarse- and fine-scale predictions; instead, the coarse branch contributes a geometric prior through *A*, while the final modulation strength is adaptively learned by ΔC, and the fine-scale branch remains the main estimator. This mechanism assigns higher weights to geometrically reliable regions and stabilizes cost modeling, improving matching robustness and boundary consistency.

### 2.2. ConvNeXtV2-Style 2D Cost Aggregation Block

**Figure 3:**
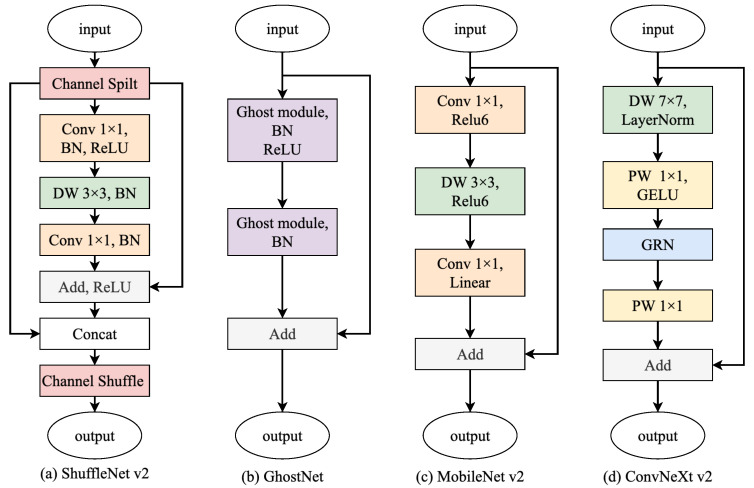
Comparison of different 2D cost aggregation blocks (e.g., ShuffleNetV2, GhostNet, MobileNetV2, ConvNeXtV2-style). In this figure, DWConv denotes depthwise convolution, LN denotes LayerNorm, GELU denotes Gaussian Error Linear Unit activation [17], and GRN denotes Global Response Normalization.

Prior 2D cost aggregation for lightweight stereo models often relies on depthwise separable convolutions and inverted residual blocks (e.g., the MobileNet family), as exemplified by MobileNetStereo-2D and LightStereo [15]. While such structures reduce computation, purely local convolutions can be limited in capturing long-range spatial dependencies and complex geometric relationships, leading to weaker aggregation representations. Here, we introduce a lightweight ConvNeXtV2-style residual block [16] into the 2D cost aggregation network. With a coordinated design of large-kernel depthwise convolution, normalization, and nonlinearity, the block enhances spatial/channel expressiveness while keeping the overall complexity controlled. A schematic comparison is shown in Figure 3.

Given an input cost feature map *X*, the block first applies a depthwise convolution to enlarge the receptive field:

(3) $$X_{1}={\mathrm{DWConv}}_{7\times 7}\left(X\right)$$

Then a 2D LayerNorm stabilizes training:

(4) $$X_{2}=\mathrm{LN}\left(X_{1}\right)$$

Next, a pointwise convolution expands channels followed by a GELU activation [17]:

(5) $$X_{3}=\mathrm{GELU}\left(W_{1}*X_{2}\right)$$

Here, ∗ denotes linear convolution rather than element-wise multiplication. We adopt GELU following the ConvNeXtV2-style design [16] because its smooth nonlinearity preserves weak responses better than a hard-threshold activation and empirically stabilizes lightweight cost aggregation. To further modulate global channel responses, a Global Response Normalization (GRN) module is applied:

(6) $$X_{4}=\mathrm{GRN}\left(X_{3}\right)$$

where GRN denotes Global Response Normalization. The channels are then projected back to the original dimension:

(7) $$X_{5}=W_{2}*X_{4}$$

Finally, if input and output dimensions match, a residual connection is used:

(8) $$Y=X+X_{5}$$

### 2.3. Overall Network Architecture

HAGVNet consists of five components: feature extraction, cost volume construction, cost aggregation, disparity regression, and the loss function.

#### 2.3.1. Multi-Scale Feature Extraction

Let the rectified left/right images be $I_{L}$ and $I_{R}$. We use an ImageNet-pre-trained MobileNetV2 [18,19] backbone to extract multi-scale features via progressive downsampling. On top of the backbone, a bidirectional feature pyramid network (BiFPN) [20] fuses information across resolutions to improve multi-scale geometric consistency. Features at 1/4 and 1/8 resolution are used for fine- and coarse-scale cost volume construction, respectively.

#### 2.3.2. Cost Volume Construction

Let $F_{L}$ and $F_{R}$ denote left and right features. At 1/4 resolution, we define the disparity hypothesis set as $D=\{0,\ldots ,D_{\max}-1\}$ with $D_{\max}=192$. For each disparity d∈D, the right feature is shifted horizontally by *d* pixels, and its similarity to the left feature is computed to form the cost volume:

(9) $$C\left(x,y,d\right)=\frac{1}{N_{s}}\langle F_{L}\left(x,y\right),F_{R}\left(x-d,y\right)\rangle$$

where 〈·,·〉 denotes the channel-wise inner product, and $N_{s}$ is the number of feature channels used for correlation normalization. The normalization term keeps the correlation magnitude comparable across feature dimensions. The fine-scale cost volume is constructed at 1/4 resolution and the coarse-scale one at 1/8 resolution, and the HAGV mechanism is used to obtain a geometry-modulated cost representation.

#### 2.3.3. Cost Aggregation

**Figure 4:**
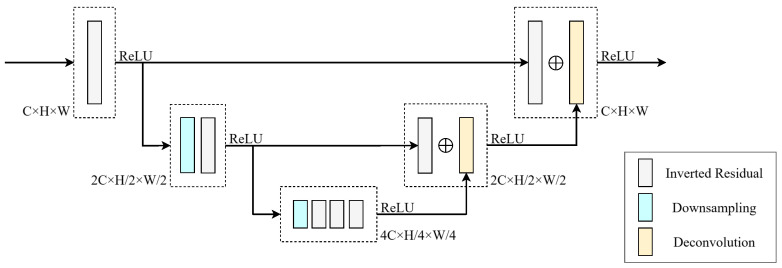
Cost aggregation structure used for coarse- and fine-scale cost volumes.

Coarse- and fine-scale cost volumes are aggregated by similar ConvNeXtV2-style residual blocks in a hierarchical manner. Each branch performs progressive downsampling (e.g., 1/2 and 1/4) and U-Net-style symmetric upsampling with skip connections to fuse multi-level information, improving spatial consistency and geometric correlation. The aggregation structure is illustrated in Figure 4.

#### 2.3.4. Disparity Regression

After cost aggregation, a soft-argmax operation is applied to the cost volume to predict the final disparity map $\hat{d}$. Using the disparity hypothesis set *D* defined above, for each pixel location (x,y), a softmax normalization is performed over C(x,y,d) along the disparity dimension *d*, yielding a disparity probability distribution:

(10) $$p\left(d|x,y\right)=\frac{\exp\;\left(-C(x,y,d)\right)}{\sum_{k=0}^{D_{\max}-1}\exp\;\left(-C(x,y,k)\right)}$$

Then the expected disparity is computed as a weighted sum:

(11) $$\hat{d}\left(x,y\right)=\sum_{d=0}^{D_{\max}-1}d\cdot p\left(d|x,y\right)$$

#### 2.3.5. Loss Function

For a single-scale disparity prediction, we use a Smooth-$\ell_{1}$ loss between the predicted disparity and ground truth over valid pixels:

(12) $$L\left(\hat{d},d^{*}\right)=\frac{1}{N}\sum_{i=1}^{N}\mathrm{SmoothL}1\left({\hat{d}}_{i}-d_{i}^{*}\right)$$

In addition, we use joint supervision on the coarse-scale and final-scale predictions to improve training stability and geometric consistency:

(13) $$L_{\mathrm{total}}=L\left(\hat{d},d^{*}\right)+\lambda \;L\left(d_{\mathrm{coarse}},d^{*}\right)$$

In all training stages, we set λ=0.2.

## 3. Obstacle Detection and Range Estimation

### 3.1. YOLO11n Detection Front-End

To obtain obstacle categories and their 2D locations in the image plane, we use YOLO11n as the front-end perception module. Given an input image *I*, YOLO11n outputs a set of detections:

(14) $$B=\{\left(b_{i},c_{i},s_{i}\right)|i=1,2,\ldots ,N\}$$

Each detection provides a 2D bounding box $b_{i}$, a class label $c_{i}$, and a confidence score $s_{i}$ for i=1,…,N. These bounding boxes are used to extract regions of interest (ROIs) from the depth map for subsequent ranging and 3D localization. Considering embedded resource and latency constraints, we adopt YOLO11n [14] as the detection model due to its small parameter count and low computational complexity while maintaining sufficient detection accuracy for obstacle perception.

### 3.2. Depth Extraction and 3D Localization

After obtaining 2D detections, stereo depth is used to estimate the metric distance and 3D position of each target. For a 3D point, let its corresponding pixel coordinates in the left and right images be $\left(u_{L},v\right)$ and $\left(u_{R},v\right)$, respectively. The horizontal disparity is $d=u_{L}-u_{R}$, and depth is given by the standard stereo relationship:

(15) $$Z=\frac{f\cdot B}{d}$$

where *f* is the effective focal length, and *B* is the stereo baseline. Thus, the disparity map predicted by HAGVNet can be converted to a dense depth map.

**Figure 5:**
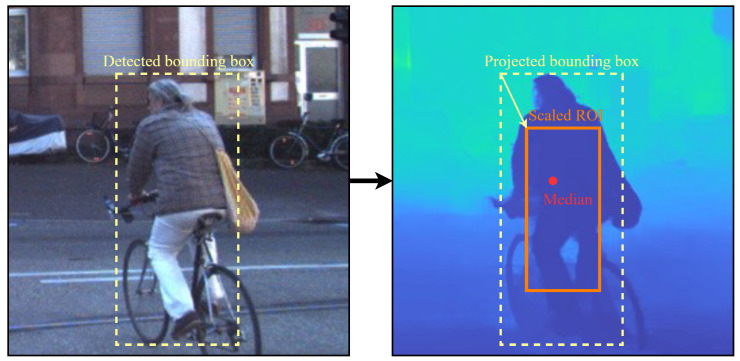
Depth extraction constrained by object detection and ROI statistics. ROI denotes region of interest; center-shrunk ROI is used to suppress boundary noise and background leakage before median depth estimation.

For robust target depth estimation, we extract an ROI in the depth map using the detected bounding box. To reduce background interference and unstable disparity near object boundaries, the bounding box is uniformly shrunk to 50% of its original width and height, and depth statistics are computed only within the shrunk ROI. To mitigate outliers, we use the median depth within the ROI as the final distance estimate. We do not rely on detector-provided segmentation masks in the current system because the bounding-box-only pipeline is simpler to deploy on embedded hardware, incurs lower latency and memory overhead, and remains more stable for thin or distant objects whose masks can be fragmented. Figure 5 illustrates the workflow.

Given the estimated depth *Z* and camera intrinsics, the 3D position of the target center in the camera coordinate system can be recovered:

(16) $$X_{i}=\frac{\left(u_{i}-c_{x}\right)\cdot Z_{i}}{f},\;Y_{i}=\frac{\left(v_{i}-c_{y}\right)\cdot Z_{i}}{f},\;Z_{i}=Z_{i}$$

The system output for each obstacle can be represented as follows:

(17) $$O_{i}=\left(c_{i},s_{i},X_{i},Y_{i},Z_{i}\right)$$

where $\left(u_{i},v_{i}\right)$ denotes the image coordinate of the target center, $\left(c_{x},c_{y}\right)$ denotes the principal point, *f* is the focal length, and $\left(X_{i},Y_{i},Z_{i}\right)$ is the recovered 3D position in the camera coordinate system. The tuple $O_{i}$ therefore contains the detected class, confidence, and target position.

## 4. Experiments

### 4.1. Datasets, Metrics, and Implementation Details

We primarily evaluate the proposed method on stereo matching benchmarks and additionally assess its deployment-oriented ranging performance. YOLO11n is trained only as a front-end component for pipeline deployment. All experiments are implemented in PyTorch and trained on a single NVIDIA RTX 4090 GPU. The software stack is PyTorch 2.6.0 with CUDA 12.6 and cuDNN 9.5 for training and TensorRT 10.3.0 (FP16) for embedded deployment.

For stereo matching, the model is first pre-trained on the SceneFlow dataset, which provides dense ground-truth disparity. End-point error (EPE) is used as the evaluation metric [21]. During pre-training, the batch size is 12, the optimizer is AdamW, and OneCycleLR is used with a maximum learning rate of $1\times 10^{-4}$ for 90 epochs. Random cropping is applied as the main augmentation. The model is then jointly fine-tuned on the union of the training splits of KITTI 2012 [22] and KITTI 2015 [23]. KITTI 2012 is evaluated using EPE, while KITTI 2015 uses D1-all (the percentage of pixels with an error greater than 3 pixels or a relative error greater than 5%). Fine-tuning runs for 500 epochs with batch size 2, OneCycleLR with maximum learning rate $2\times 10^{-5}$, and augmentations including color jitter, random erasing, random scaling, and random cropping.

For object detection, we train YOLO11n using COCO [24] and VIDVIP [25]. YOLO11n is used only as a supporting front-end component rather than for method-to-method comparison; its outputs are used for ranging and deployment-oriented validation.

### 4.2. Stereo Matching Experiments and Analysis

To evaluate the accuracy–complexity trade-off, we address three topics: (Q1) which lightweight convolution block provides the strongest baseline; (Q2) how much accuracy gain HAGV brings relative to its overhead; and (Q3) where HAGVNet lies relative to representative real-time/lightweight methods. Since latency/FPS values in prior work depend strongly on hardware and deployment stacks, we do not make direct cross-paper speed claims. Instead, we use FLOPs to characterize complexity trends rather than absolute runtime equivalence. FLOPs are computed as multiply–add operations in a single forward pass (for convolution layers, $\mathrm{FLOPs}=2\times H\times W\times C_{\mathrm{in}}\times C_{\mathrm{out}}\times k_{h}\times k_{w}/\mathrm{groups}$, where *H* and *W* are the spatial height and width, $C_{\mathrm{in}}$ and $C_{\mathrm{out}}$ are the input and output channel numbers, $k_{h}$ and $k_{w}$ are the kernel height and width, and groups is the convolution group number); our model is measured with this convention, while baseline FLOPs are reported based on the original papers.

#### 4.2.1. Ablation on Convolution Blocks

Under the same network structure and training strategy, we compare different convolution blocks on SceneFlow, as summarized in Table 1. ConvNeXtV2 [16] achieves the lowest EPE (0.7644). Relative to MobileNetV2 [18], the EPE is lower by 0.0291 (0.7935 → 0.7644, about 3.7%), while FLOPs are lower by 0.74 G (20.32 → 19.58 G). Relative to GhostNet [26] and ShuffleNetV2 [27], the EPE is lower by 0.0627 (about 7.6%) and 0.1579 (about 17.1%), respectively, with moderate complexity differences across the compared blocks (17.93–20.32 G FLOPs and 2.65–3.44 M parameters). These results indicate that ConvNeXtV2 delivers stable accuracy gains at acceptable computational cost and is selected as the base aggregation unit in subsequent experiments.

#### 4.2.2. Ablation on the HAGV Module

To verify the effectiveness of the proposed HAGV module, we introduce HAGV (with different coarse-scale settings) on top of the ConvNeXtV2-based baseline, as reported in Table 2. Using only 1/4-resolution features for cost modeling, the baseline reaches 0.7644 EPE on SceneFlow. Adding a 1/16 coarse branch reduces the EPE to 0.7612 (lower by 0.0032, about 0.4%), while FLOPs increase from 19.58 G to 19.61 G (higher by 0.03 G, about 0.15%). Using a 1/8 coarse branch reduces the EPE to 0.7415 (lower by 0.0229, about 3.0%) while FLOPs increase to 20.08 G (higher by 0.50 G, about 2.6%). These results indicate that coarse-scale geometric priors improve matching stability and that 1/8 provides a better accuracy–complexity trade-off.

#### 4.2.3. Comparison with Representative Methods

We further compare HAGVNet with representative real-time/lightweight stereo methods on SceneFlow [21] and KITTI [22,23]. Among these baselines, StereoNet, AANet, and HITNet also use coarse-to-fine or multi-scale reasoning, but they do not use a coarse-scale geometric prior to explicitly modulate the fine-scale cost volume in the same way as HAGV. The KITTI 2012 and KITTI 2015 test results are obtained by separate submissions to their official evaluation servers.

**Figure 6:**
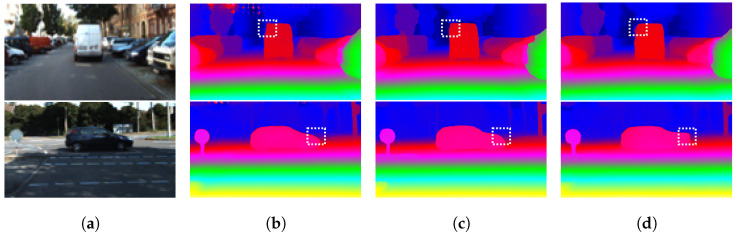
Qualitative comparison of HAGVNet with representative methods on KITTI. (**a**) Left image. (**b**) HAGVNet (Ours). (**c**) MobileStereoNet-2D. (**d**) HITNet.

**Figure 7:**
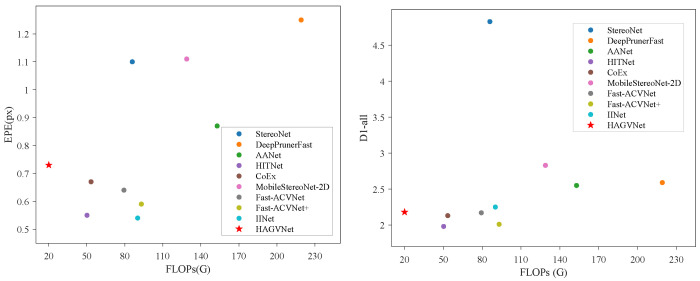
Accuracy vs. FLOP comparison on SceneFlow (**left**) and KITTI 2015 (**right**).

Detailed quantitative comparisons are presented in Table 3 (SceneFlow) and Table 4 (KITTI). On SceneFlow, HAGVNet reaches 0.73 px EPE with 20.08 G FLOPs, which is the lowest reported FLOP value among the listed methods in Table 3. On KITTI 2015, HAGVNet achieves D1-all = 2.18, which is lower by 0.37/0.41/0.65/2.65/0.07 compared to AANet [9], DeepPrunerFast [28], MobileStereoNet-2D [10], StereoNet [8], and IINet [13], respectively. Compared with HITNet [11], Fast-ACVNet [12], and Fast-ACVNet+ [12], D1-all is higher by 0.20/0.01/0.17, while reported FLOPs are lower from 50.23/79.34/93.08 G to 20.08 G. These results indicate that under a low computation budget, HAGVNet offers a competitive Pareto trade-off rather than the absolute best accuracy. As shown in Figure 6, HAGVNet also preserves structure continuity and suppresses local matching noise in weak-texture and large planar regions. Figure 7 shows the accuracy–FLOP trade-off on SceneFlow and KITTI 2015.

### 4.3. Obstacle Detection and Range Estimation Experiments

#### 4.3.1. Platform and Stereo Camera Configuration

**Figure 8:**
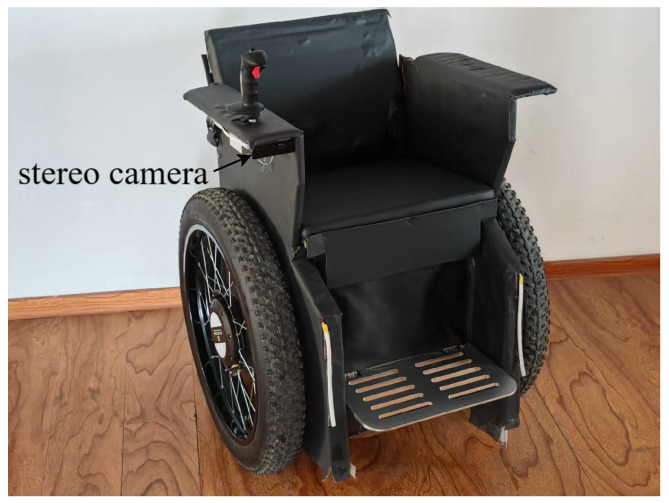
Micro-mobility vehicle platform and stereo camera installation.

We use a Jetson Orin Nano Super (8 GB) as the embedded computation unit for perception and depth estimation. The stereo sensor is configured at 640×480 resolution and 60 FPS, with a 7.5 cm baseline and an approximately 89.5° field of view. The installation setup is shown in Figure 8.

#### 4.3.2. Deployment and Inference Performance

We further evaluate representative stereo matchers under a unified embedded deployment setting on Jetson Orin Nano Super at an input resolution of 640×480, using the same timing protocol for both PyTorch and TensorRT FP16 inference. As summarized in Table 5, HAGVNet has a computational complexity of 20.08 G FLOPs, and under the present test setting, its measured runtime decreases from 101.6 to 21.6 ms after TensorRT acceleration, corresponding to 46.3 FPS. The unified on-device results indicate that although lower FLOPs cannot be treated as directly equivalent to higher FPS or lower latency across different models and deployment stacks, they do reflect an efficiency trend and can serve as a useful reference when selecting models for embedded deployment. In the present setting, this trend is consistent with the measured runtime ordering in Table 5. YOLO11n is used only as a lightweight front-end detector for ROI extraction in the complete ranging pipeline; under the same TensorRT FP16 setting, its measured latency is 6.9 ms. In full pipeline deployment (detection + stereo matching + depth fusion ranging) at an input resolution of 640×480, the measured end-to-end refresh rate is 30 Hz; since the detector latency is substantially lower than the stereo matching latency, the primary latency bottleneck remains stereo matching.

#### 4.3.3. Field Ranging Experiments

After deployment, we conduct field tests in a park environment with representative obstacles (e.g., pedestrians, vehicles, street lamps, and static facilities) at varying distances. Ground-truth distances from the camera center to the targets are measured using a handheld laser rangefinder with a nominal accuracy of ±3mm. For consistency, the measurement point is chosen at the geometric center of the visible target surface. These measured distances are used as reference values to evaluate the stereo ranging outputs.

**Figure 9:**
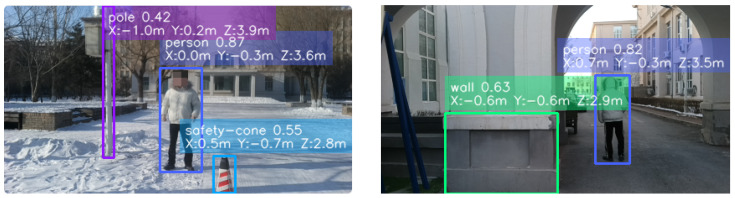
Visualization of field ranging results.

As shown in Figure 9, the deployed pipeline provides stable target-level ranging results under complex backgrounds. As summarized in Table 6, each category is evaluated at five distances (2–10 m), and the overall mean relative error across all 30 measurements is about 3.52%. The mean relative errors by distance are about 1.42% (2 m), 2.53% (4 m), 2.72% (6 m), 4.57% (8 m), and 6.38% (10 m), showing increasing error with distance as disparity resolution decreases at longer range. We additionally observe larger error fluctuations on hollow objects (e.g., an unoccupied motorcycle). A likely cause is the ROI median strategy together with the current point selection rule: the shrunk ROI may include background depth through central cavities, which perturbs the representative depth value.

## 5. Conclusions

This paper targets micro-mobility vehicles in closed, low-speed environments and focuses on the design and deployment validation of a lightweight stereo matching method for target-level ranging. To achieve this goal, HAGVNet introduces coarse-scale geometric priors to modulate fine-scale cost modeling, while ConvNeXtV2-style 2D cost aggregation improves disparity stability with low computational overhead. On public benchmarks, HAGVNet reaches 0.73 px EPE on SceneFlow with 20.08 G FLOPs and achieves 2.18 D1-all on KITTI 2015, indicating a competitive Pareto trade-off under low computation budgets. On a Jetson Orin Nano Super, TensorRT (FP16) deployment enables 46.3 FPS real-time inference for HAGVNet, while the full perception pipeline runs at 30 Hz end-to-end at 640×480 input resolution. These results further support the suitability of the proposed stereo matching model for lightweight deployment scenarios, where achieving competitive accuracy under low computational complexity is of primary importance. Combined with a YOLO11n front-end, field tests achieve an overall mean relative ranging error of about 3.52% and a maximum relative error of 8.6% within 2–10 m, demonstrating that the proposed lightweight stereo module can provide reliable depth cues for practical target-level ranging in low-speed obstacle avoidance scenarios. Future work should include improving robustness under weak texture, strong illumination, and occlusion; enhancing cross-scene adaptation; and leveraging temporal cues and online calibration for long-term stability.

## Figures and Tables

**Table 1:** Ablation study of different convolution blocks on SceneFlow [21].

Block	FLOPs (G)	Params (M)	EPE
GhostNet [26]	17.94	2.82	0.8271
ShuffleNetV2 [27]	17.93	2.65	0.9223
MobileNetV2 [18]	20.32	3.44	0.7935
ConvNeXtV2 [16]	19.58	3.40	0.7644

**Table 2:** Ablation study of HAGV module on SceneFlow [21].

	Coarse	Fine	FLOPs (G)	Params (M)	EPE
Baseline	-	1/4	19.58	3.40	0.7644
+HAGV	1/16	1/4	19.61	3.50	0.7612
+HAGV	1/8	1/4	20.08	3.78	0.7415

**Table 3:** Comparison with representative real-time stereo matching networks on SceneFlow.

Method	FLOPs (G)	Params (M)	EPE
StereoNet [8]	85.93	0.40	1.10
AANet [9]	219.12	7.47	1.25
DeepPrunerFast [28]	152.86	2.97	0.87
HITNet [11]	50.23	0.42	0.55
CoEx [29]	53.39	2.72	0.67
MobileStereoNet-2D [10]	128.84	2.23	1.11
Fast-ACVNet [12]	79.34	3.08	0.64
Fast-ACVNet+ [12]	93.08	3.20	0.59
IINet [13]	90.16	19.54	0.54
HAGVNet (Ours)	20.08	3.78	0.73

**Table 4:** Comparison with representative lightweight stereo matching networks on KITTI.

Method	KITTI 2012		KITTI 2015					FLOPs (G)
	3-noc	3-all	4-noc	4-all	D1-bg	D1-fg	D1-all	
StereoNet [8]	–	–	–	–	4.30	7.45	4.83	85.93
DeepPrunerFast [28]	–	–	–	–	2.32	3.91	2.59	219.12
AANet [9]	1.91	2.42	1.46	1.87	1.99	5.39	2.55	152.86
HITNet [11]	1.41	1.89	1.14	1.53	1.74	3.20	1.98	50.23
CoEx [29]	1.55	1.93	1.15	1.42	1.79	3.82	2.13	53.39
MobileStereoNet-2D [10]	–	–	–	–	2.49	4.53	2.83	128.84
Fast-ACVNet [12]	1.68	2.13	1.23	1.56	1.82	3.93	2.17	79.34
Fast-ACVNet+ [12]	1.45	1.85	1.06	1.36	1.70	3.53	2.01	93.08
IINet [13]	1.81	2.21	1.35	1.65	2.02	3.39	2.25	90.16
HAGVNet (Ours)	1.87	2.27	1.32	1.62	1.86	3.76	2.18	20.08

**Table 5:** Unified on-device deployment comparison of representative stereo matchers on Jetson Orin Nano Super at 640×480 input resolution.

Method	FLOPs (G)	PyTorch (ms)	TRT (ms)	TRT (FPS)
MobileStereoNet-2D	128.84	465.8	165.1	6.1
CoEx	53.39	120.4	63.7	15.7
HAGVNet (Ours)	20.08	101.6	21.6	46.3

**Table 6:** Field ranging results by obstacle category (five distances per category).

ID	Obstacle	2 m	4 m	6 m	8 m	10 m
A1	Pedestrian	2.02 (1.0%)	3.93 (1.8%)	6.09 (1.5%)	8.46 (5.8%)	10.74 (7.4%)
A2	Car	1.99 (0.5%)	4.03 (0.8%)	5.93 (1.2%)	8.11 (1.4%)	10.52 (5.2%)
A3	Motorcycle	1.93 (3.5%)	4.19 (4.8%)	5.71 (4.8%)	8.40 (5.0%)	9.19 (8.1%)
A4	Roadblock	2.04 (2.0%)	3.88 (3.0%)	6.15 (2.5%)	8.43 (5.4%)	10.86 (8.6%)
A5	Pole	1.98 (1.0%)	4.13 (3.3%)	5.67 (5.5%)	8.44 (5.5%)	9.53 (4.7%)
A6	Tree	2.01 (0.5%)	3.94 (1.5%)	6.05 (0.8%)	8.34 (4.3%)	10.43 (4.3%)

## Data Availability

Publicly available datasets were analyzed in this study. The Scene Flow dataset is available at https://lmb.informatik.uni-freiburg.de/resources/datasets/SceneFlowDatasets.en.html (accessed on 10 February 2026), the KITTI dataset is available at https://www.cvlibs.net/datasets/kitti/ (accessed on 10 February 2026), the COCO dataset is available at https://cocodataset.org/ (accessed on 10 February 2026), and the VIDVIP dataset is described and made available by its authors at https://doi.org/10.20965/jrm.2021.p1135 (accessed on 10 February 2026). Additional processed data supporting the findings of this study are available from the corresponding author upon reasonable request.

## Abbreviations

The following abbreviations are used in this manuscript:

HAGV	Hierarchical attention-guided cost volume
BiFPN	Bidirectional feature pyramid network
GRN	Global response normalization
GELU	Gaussian error linear unit
ROI	Region of interest
FLOPs	Floating-point operations
EPE	End-point error
FPS	Frames per second
